# The evolution of the subjective well-being of the general population during the COVID-19 pandemic period: the case of Belgium

**DOI:** 10.1038/s41598-023-46824-3

**Published:** 2023-11-16

**Authors:** Sara Claes, Sophie Vandepitte, Lieven Annemans

**Affiliations:** Faculty of Medicine and Health Sciences, Department of Public Health and Primary Care, Interuniversity Centre for Health Economics Research (I-CHER), Ghent University, Ghent, Belgium

**Keywords:** Psychology, Public health

## Abstract

The consequences of the COVID-19 pandemic period on a nation’s well-being remain inadequately understood, especially over time. Therefore, this research aims to map the changes in the subjective well-being (SWB) of the general Belgian adult population during the COVID-19 pandemic. Analyses of variances (ANOVA) were performed to evaluate the changes in SWB during the pandemic at five different time points during the period from February 2020 until December 2022 using repeated cross-sectional representative samples of the Belgian population. The differences over time in subjective well-being were investigated in terms of life evaluation, positive affect, and negative affect. The changes in autonomy, competence, relatedness, loneliness and peace of mind were also explored as explanatory variables. Moderation analyses were performed to investigate the differential changes in well-being for different age groups. Our results show the subjective well-being of the Belgian population decreased during the COVID-19 pandemic, notably during the second lockdown and the fifth wave. Furthermore, younger individuals are significantly more susceptible to negative changes in well-being during the pandemic period. Finally, our results show that peace of mind is an important predictor of all SWB components during the pandemic. Based on these results several policy recommendations are formulated.

## Introduction

The COVID-19 crisis has an unseen global impact on our society^1^, with drastic policy measures being taken during the peak of the pandemic to contain the virus transmission. These policy measures included limiting social contact, mandatory face mask use, closure of schools and non-essential business activities, banning of public gatherings, closing country borders, and a countrywide lockdown^2^. Focusing on preventing the spread of the virus, these policy measures aimed to lower the pandemic’s burden, and to prevent a healthcare system collapse by protecting individuals’ physical health^3,4^.

Physical health definitely is a precarious basic need. However, the consequences on other life domains by protecting this precarious need are enormous. The COVID-19 crisis has indeed heightened the risk factors generally associated with poor mental health (e.g. financial insecurity, unemployment, fear), while protective factors (e.g. social connection, daily routine, employment and educational engagement, access to physical exercise and to health services) fell dramatically^5^. Early evidence from the beginning of the COVID-19 pandemic indeed shows that lockdown measures put citizens, and especially vulnerable groups, at risk of developing several mental health problems, such as depression and anxiety^6,7^. Several characteristics of individuals that are particularly vulnerable for mental health problems and lower well-being during lockdowns were identified. These include loss of income, decreased access to basic supplies, being female, pre-existing psychological disorders, lower self-rated health, and relatives diagnosed with COVID-19^6,8^. However, lockdowns do not only appear to be harmful for the well-being of such vulnerable individuals. The study of Fiorillo et al.^9^ for instance concluded, based on the data of 20,720 people, that lockdowns pose a serious threat for the general population’s mental health. Also, a review by Vindegaard and Benros^8^ found that the general public showed lower psychological well-being and higher scores of anxiety and depression compared to before COVID-19. Similar conclusions can be drawn from a recent review on mental health during the first year of the COVID-19 pandemic^10^. These authors revealed that anxiety, depression and distress increased during the early months of the pandemic.

This diminished mental well-being can be related to several aspects of the pandemic and its related policy measures. First of all, people experienced sudden tremendous changes in their daily routines, and social and leisure activities. Several situations, such as social interactions, suddenly changed from positive to potentially dangerous^6^. Moreover, due to lockdown measures many people have (temporary) lost their income which also impairs mental health^6,11^. Also, the substantial reduction of daily activities as a result of lockdowns may have caused severe boredom and reduced reinforcement known to be associated with depression^12^. However, not everyone suffered equally and substantial variations existed in how people might experience these lockdowns. For instance, differences existed in the degree to which individuals were confined as some people were still allowed to go to work, whereas others became very isolated and even experienced difficulties in obtaining basic supplies.

All of these lockdown-specific features may have impacted one’s mental health to a different extent during the different waves of the COVID-19 pandemic. However, to date the magnitude of these consequences on the overall nations’ well-being remains inadequately explored, especially over time. The longitudinal studies to date that examine the impact of COVID-19 and its policy measures on well-being occurred only during the first year of the pandemic, limiting our understanding of the long-term impact of the pandemic on a nation’s overall well-being^8,10,13–16^.

Subjective well-being is increasingly being reported in addition to objective economic data to investigate social progress and prosperity, to evaluate public policy, and to predict outcomes in individuals and societies^17–19^. Moreover, subjective well-being has been identified as a key aspect in evaluating psychosocial impact within health emergency contexts, such as the COVID-19 pandemic. Therefore and in line with the call of Aknin et al.^10^, we aim to estimate the long-term changes in subjective well-being (SWB) during the COVID-19 pandemic and thereby providing a guideline to tackle them. Moreover, the value of studying this broad concept in the light of this pandemic becomes even more clear when consulting Ed Diener’s definition^20^. This definition states that SWB has both an evaluative and affective dimension. As such, high SWB can be defined as a reflection of positive emotions and thoughts about life in terms of frequent positive affect and infrequent negative affect (the affective dimension) and a sense of high satisfaction with life as a whole (the evaluative dimension)^20,21^.

This study aims to map the changes in a nation’s overall SWB over time during the COVID-19 pandemic and its associated lockdowns. In particular, the changes in the SWB of Belgian citizens was investigated at five different time points during the period from February 2020 until December 2022 based on repeated cross-sectional representative samples of the Belgian population. At these time points, not only the changes in positive and negative affect and in life evaluation as a whole were measured^20,22^, but also the evolution of several other SWB indicators, such as: autonomy, relatedness, competence, loneliness, and peace of mind^23^—of which the last one was recently recognized as an important determinant of mental and general well-being^24,25^. Next, we investigate how these well-being indicators relate to SWB in times of a pandemic, to further explain the changes in SWB during the COVID-19 pandemic. Furthermore, we investigate whether changes in well-being during the pandemic period differ for individuals from different age groups, since previous findings show a larger increase in psychological distress for younger individuals^10^. The results of this study will inform policymakers on the long-term consequences of the pandemic period and policy measures taken to control the virus transmission on a nation’s overall well-being. Moreover, our results will have international value in the sense that this unique national large-scale repeated cross-sectional study indeed allows to compare SWB before the COVID-19 pandemic and afterwards. As many countries are facing the same challenges while implementing similar policy measures, the Belgian results will also be of important value for their policy strategies. No doubt that nations all over the globe will be facing a challenge to deal with the consequences of the pandemic period and in restoring and improving financial and social prosperity^26^. The insights from this study can then be used by policymakers as a guideline to determine the best policy strategy in tackling the most urgent problems, whilst also protecting prosperity and well-being in their nations in the short and long term.

## Methods

**Figure 1:**
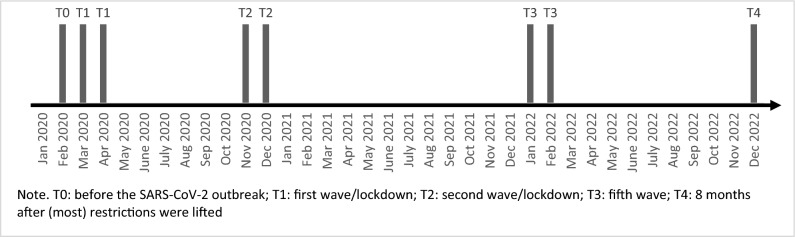
Overview of measurement points.

The present study uses the cross-sectional data based on a convenience sample of the Belgian National NN-UGhent happiness study. This study was held at various time points during the period from February 2020 until December 2022 (see Fig. 1). At all timepoints, a cross-sectional sample of the Belgian population completed an online anonymous questionnaire after obtaining approval from the Ethical Committee of the Ghent University Hospital. The study procedures were performed in accordance with relevant guidelines and regulations. Before entering the study, informed consent of the participants was obtained. As such, data of Belgians have been collected *before the SARS-CoV-2 outbreak (February 2020) (T0), during the first wave/lockdown (March/April 2020) (T1)*, during the *second wave/lockdown (November/December 2020) (T2)*, *during the fifth wave (January/February 2022) (T3),* and *8 months after (most) COVID-19 restrictions were lifted (December 2022) (T4)* to evaluate the longer term changes in well-being during this pandemic. At each timepoint, a representative subsample of respectively N = 1466, N = 1452, N = 1380, N = 1602 and N = 1709 was created using sample weights. The resulting samples were all representative for age, gender, educational level, professional status and region. Inclusion criteria to participate were living in Belgium and sufficient understanding of Dutch or French. An overview of the policy measures in place for each time point are presented in Table 1.

**Table 1:** Overview of COVID-19 related policy measures in Belgium at each time point.

T0 (February 2020): before the outbreak	No restrictions
T1 (March/April 2020): first wave	First country-wide lockdown which included Closure of schools and non-essential business activities, Delay of non-essential medical care, Banning of public gatherings, And closing country borders Face mask use was not mandatory
T2 (November/December 2020): second wave	Second country-wide lockdown which included: Closure of non-essential business activities (until December 2020), Closure of hospitality sector and non-medical contact professions, Delay of non-essential medical care, Mandatory face mask use for pupils ≥ 12y, closure of universities (distance learning), Mandatory telework, Banning of public gatherings, and limiting social contacts (max 1–3 persons allowed in social “bubble”) Mandatory face mask use, Mandatory testing in case of high risk contact or return form “red” zone
T3 (January/February 2022): fifth wave	Policy measures included: Mandatory closing hour hospitality sector and closure night clubs (until February 18th), Mandatory face mask use for all individuals ≥ 6y, Banning of indoor activities (except sports), Limiting indoor public gatherings to 200 attendees
T4 (December 2022): 8 months after most restrictions were lifted	Policy measures included: Mandatory face mask use in healthcare institutions
Based on^27^	

## Measures

### Components of subjective well-being

*Life evaluation* (the evaluative component of SWB) was measured with the Cantril Ladder^22,28,29^ which asks the following question: “Please imagine a ladder with steps numbered from zero at the bottom to ten at the top. The top of the ladder represents the best possible life for you and the bottom of the ladder represents the worst possible life for you. On which step of the ladder do you feel you personally stand at the present time?”^30^. This one-item question is considered reliable as a correlation of 0.75 between the Cantril Ladder from the Gallup World Poll and life satisfaction as measured in the World Values Survey was found^28^.

*Positive and Negative affect* (the affective component of SWB) were questioned using an abbreviated version of the Positive and Negative Affect Schedules (PANAS)^31^. Positive affect was the sum of five positive emotions items divided by the number of items, while negative affect was the sum of five negative emotions divided by the number of items. This total score was rescaled to a score with a range between 0 and 10. The Cronbach’s alpha was 0.717 in the negative affect scale and 0.757 in the positive affect scale.

### Explanatory variables

To further explain the changes in subjective well-being during the COVID-19 pandemic, we investigated the role of several explanatory variables: basic psychological needs (autonomy, competence, and relatedness), loneliness, and peace of mind.

*Basic psychological needs* The Self-Determination Theory identifies three basic intrinsic psychological needs that are essential for the well-being, the intrinsic motivation and the self-regulation of individuals: autonomy (a sense of psychological freedom), competence (sense of effectiveness and mastery) and relatedness (sense of connection with important others). Fulfillment of these three innate psychological needs leads to enhanced well-being, creativity and performance, while a lack of fulfillment causes diminished motivation and ill-being^32^. To assess these basic psychological needs a shortened version of the Basic Psychological Need Satisfaction and Frustration Scale (12 items)^33^ was used. This scale combines a balanced combination of satisfaction (positively formulated) and frustration (negatively formulated) items^34^. Each psychological need was evaluated by combining positive and negative items on a 5-point Likert scale ranging between 0 (never) and 4 (always). Scores for each psychological need were then calculated by subtracting the positive item from the negative item of each particular need and eventually rescaled to a score with a range between 0 and 10. In our sample the Cronbach alpha scores were respectively: 0.683 for the autonomy items, 0.763 for the competence items and 0.738 for the relatedness items.

*Loneliness* was measured using the 6-item De Jong Gierveld Loneliness Scale^35^. Each item was initially scored on a Likert scale from 1 (totally disagree) to 5 (totally agree). The Cronbach alpha score for these 6 items in our sample was 0.856. Next, participants responses were rescored. When a participant’s answer to an item was indicative of loneliness a score of 1 was given; if not, a score of 0 was given for that particular item. Loneliness was then the sum of these scores and as such ranged between 0 and 6.

*Peace of mind* To collect data on the peace of mind an abbreviated version (4 items) of the Peace of Mind Scale was used^23^ containing the following statements and using a 5 point Likert scale from 1 (not at all) to 5 (all of the time): ‘my mind is free and at ease’; ‘I have peace and harmony in my mind’; ‘I feel anxious and uneasy in my mind’; ‘My lifestyle gives me feelings of peace and stability’. The total score was rescaled to a score with a range between 0 and 10. The Cronbach alpha score in our sample was 0.859.

### Sociodemographic characteristics

The representative subsamples were each weighted based on educational level, gender, region, occupational status, and age. Educational level was divided in three groups according to the International Standard Classification of Education^36^: low educated (from early childhood education to lower secondary education), medium educated (upper secondary education to post-secondary non-tertiary education), and high educated (short-cycle tertiary education to doctoral degree or equivalent). Gender was divided in three categories: woman, man, and other. The variable region distinguished between Flanders, Brussels, and Wallonia. Occupational status contained the following categories: professionally active (worker, employee, self-employed, official), unemployed, unable to work, retired (or early retirement), student, housewife/man, career break, or other situation. Four age groups were distinguished: early adulthood (ages 18–34), early middle age (ages 35–49), late middle age (ages 50–64), and late adulthood (ages 65 and older).

### Statistical analysis

Data were analyzed using IBM SPSS statistical software (version 26.0). First, descriptive statistics of the participants’ characteristics were undertaken at the five timepoints to describe the study sample. For continuous variables, mean and standard deviations were shown, while for categorical variables, percentages were used. Second, several one-way analyses of variance (ANOVA) were performed to investigate differences in the SWB outcomes over time (life evaluation, positive affect, negative affect) and in the variables that may explain SWB (autonomy, competence, relatedness, loneliness and peace of mind). Thirdly, moderation analyses were conducted to assess the differential changes in well-being during the pandemic period for different age groups. Fourth, to better understand the changes in subjective well-being (life evaluation, negative affect and positive affect), multiple regression analyses were performed for each outcome variable. Regression analyses were performed in the aggregate sample including all five timepoints. These regression models included the basic psychological needs (autonomy, competence and relatedness), loneliness, peace of mind and time point as explanatory variables and were controlled for age, gender, professional status, region, educational level and equivalised income. To test for multicollinearity, correlations and collinearity diagnostics (Variance Inflation Factor, Tolerance) were conducted^37^. We furthermore tested our regression models for autocorrelation using the Durbin-Watson test statistic which showed no indication of such autocorrelation. All analyses were conducted within the representative subsamples.

### Ethics approval and consent to participate

This study has obtained ethical approval from the ethical committee of the University of Ghent (B670201940146). All participants singed an informed consent before participation.

## Results

The participants’ characteristics are outlined in Table 2. At each time point, a representative sample of the Belgium population was created using sample weights. As such, the samples have similar characteristics at all five time points. The average age of our sample (all timepoints average) was 48.03 (SD = 15.74) and approximately half our sample identified as female (50.8%). Approximately one third of our sample was highly educated (35.6%). The majority of our sample lived in Flanders (58.5%) and was professionally active (55.5%).

**Table 2:** Characteristics of study participants.

				Continuous: Mean (SD) Categorical: n (%)	
	T0 (N = 1466)	T1 (N = 1452)	T2 (N = 1380)	T3 (N = 1602)	T4 (N = 1709)
Demographics					
Age	47.84 (16.03)	48.09 (15.83)	47.96 (16.05)	47.82 (16.21)	47.75(15.76)
Gender					
Male	715 (48.8%)	697 (48.1%)	676 (49.0%)	783 (48.9%)	840 (49.1%)
Female	742 (50.6%)	749 (51.6%)	696 (50.5%)	811 (50.6%)	865 (50.6%)
Other	5 (0.3%)	5 (0.3%)	7 (0.5%)	8 (0.5%)	4 (0.3%)
Educational level					
Low	402 (27.4%)	370 (25.5%)	372 (27.0%)	439 (27.4%)	468 (27.4%)
Middle	556 (37.9%)	514 (35.4%)	526 (38.1%)	607 (37.9%)	648 (37.9%)
High	509 (34.7%)	568 (39.1%)	482 (34.9%)	556 (34.7%)	593 (34.7%)
Region					
Flanders	850 (58.0%)	882 (60.7%)	797 (57.8%)	929 (58.0%)	991 (58.0%)
Brussels	151 (10.3%)	140 (9.6%)	143 (10.4%)	165 (10.3%)	176 (10.3%)
Wallonia	465 (31.7%)	430 (29.6%)	440 (31.9%)	508 (31.7%)	542 (31.7%)
Occupational status					
Employed	819 (55.9%)	815 (56.1%)	769 (55.7%)	877 (54.7%)	941 (55.1%)
Housewife/husband	42 (2.9%)	41 (2.8%)	54 (3.9%)	34 (2.1%)	65 (3.8%)
Unemployed	67 (4.6%)	62 (4.3%)	64 (4.6%)	72 (4.5%)	79 (4.6%)
Retired (or early retirement)	352 (24.0%)	337 (23.2%)	331 (24.0%)	400 (24.9%)	390 (22.8%)
Unable to work	80 (5.5%)	102 (7.0%)	79 (5.7%)	116 (7.2%)	117 (6.8%)
Career break	1 (0.1%)	3 (0.2%)	4 (0.3%)	9 (0.6%)	4 (0.2%)
Student	65 (4.4%)	58 (4.0%)	53 (3.8%)	72 (4.5%)	75 (4.4%)
Other	40 (2.7%)	34 (2.4%)	26 (1.9%)	23 (1.5%)	37 (2.2%)

### Changes in well-being over time

**Figure 2:**
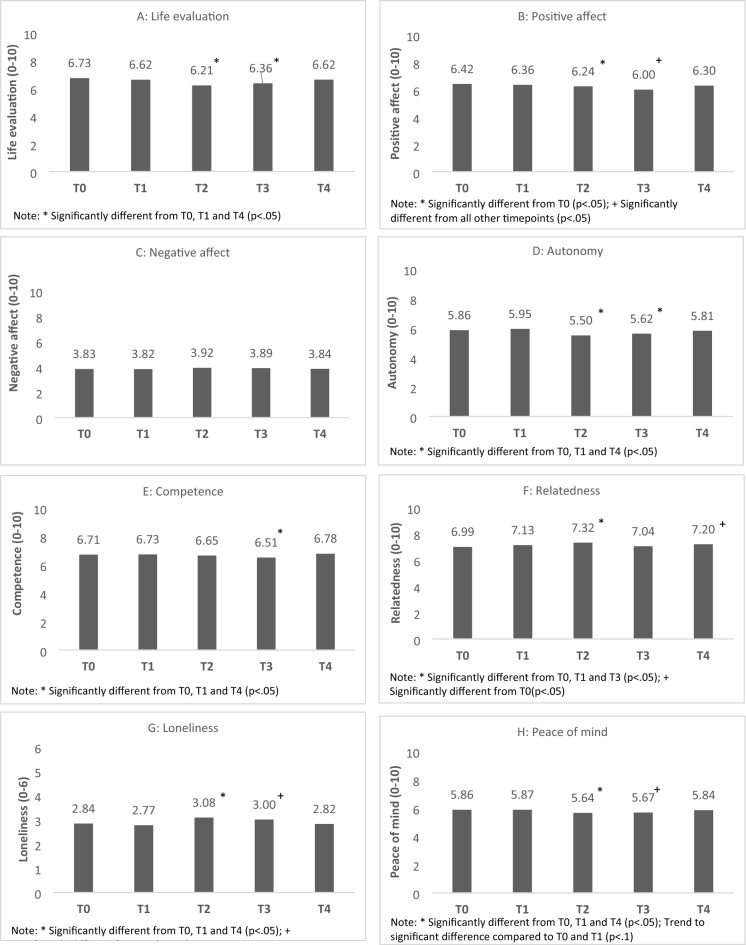
Results of one-way ANOVA [independent variable: time; dependent variables: (**a**) life evaluation, (**b**) positive affect, (**c**) negative affect, (**d**) autonomy, (**e**) competence, (**f**) relatedness, (**g**) loneliness, and (**h**) peace of mind].

Changes in all well-being indicators for the general population are shown in Table 3 and Fig. 2a–h.

**Table 3:** Evolution in well-being over time—results of one-way ANOVA.

		T0 (N = 1466)	T1 (N = 1452)	T2 (N = 1380)	T3 (N = 1602)	T4 (N = 1709)	F
Subjective well-being							
Life evaluation (0–10)	M (SD)	6.73 (1.80)^a^	6.62 (1.93)^a^	6.21 (2.12)^b^	6.36 (2.00)^b^	6.62 (1.84)^a^	F(4,7607) = 18.23***
	CI	[6.64; 6.82]	[6.52; 6.72]	[6.09; 6.32]	[6.26; 6.45]	[6.53; 6.70]	
Positive affect (0–10)	M (SD)	6.42 (1.44)^a^	6.36 (1.52)^a,b^	6.24 (1.54)^b^	6.00 (1.64)^c^	6.30 (1.52)^a,b^	F(4,7605) = 16.83***
	CI	[6.35; 6.49]	[6.28; 6.43]	[6.16; 6.32]	[5.92; 6.09]	[6.23; 6.37]	
Negative affect (0–10)	M (SD)	3.83 (1.56)	3.82 (1.61)	3.92 (1.63)	3.89 (1.74)	3.84 (1.67)	F(4,7605) = 1.08, n.s
	CI	[3.75; 3.91]	[3.74; 3.90]	[3.84; 4.00]	[3.81; 3.98]	[3.76; 3.92]	
Explanatory variables							
Autonomy (0–10)	M (SD)	5.86 (2.04)^a^	5.95 (1.95)^a^	5.49 (1.76)^b^	5.61 (1.83)^b^	5.81 (1.86)^a^	F(4,7605) = 14.68***
	CI	[5.76; 5.94]	[5.85; 6.04]	[5.40; 5.59]	[5.53; 5.71]	[5.73; 5.90]	
Competence (0–10)	M (SD)	6.71 (1.88)^a^	6.73 (1.89)^a^	6.65 (1.86)^a,b^	6.51 (1.95)^b^	6.78 (1.90)^a^	F(4,7605) = 4.92**
	CI	[6.62; 6.81]	[6.64; 6.83]	[6.56; 6.75]	[6.41; 6.61]	[6.69; 6.87]	
Relatedness (0–10)	M (SD)	6.98 (1.63)^a^	7.12 (1.69)^a,b^	7.32 (1.78)^c^	7.04 (1.81)^a,b^	7.20 (1.81)^b,c^	F(4,7605) = 8.10***
	CI	[6.91; 7.07]	[7.04; 7.21]	[7.23; 7.41]	[6.95; 7.13]	[7.11; 7.28]	
Loneliness (0–6)	M (SD)	2.84 (2.20)^a,b^	2.77 (2.11)^b^	3.08 (2.01)^c^	3.00 (2.15)^b,c^	2.82 (2.17)^a,b^	F(4,7605) = 5.73***
	CI	[2.72; 2.95]	[2.66; 2.88]	[2.98; 3.19]	[2.90; 3.11]	[2.72; 2.92]	
Peace of mind (0–10)	M (SD)	5.86 (2.04)^a^	5.86 (2.08)^a^	5.64 (2.08)^b^	5.67 (2.08)^a,b^	5.83 (2.02)^a,b^	F(4,7605) = 4.162**
	CI	[5.76; 5.97]	[5.76; 5.97]	[5.54; 5.76]	[5.57; 5.77]	[5.74; 5.94]	

Means are significantly different if they have different superscripts.

*n.s.* Not significant.

*p < .05, **p < .01, ***p < .001.

#### Life evaluation

There was a statistically significant difference in life evaluation across the five timepoints (see Table 3 and Fig. 2a). Post hoc analyses showed that life evaluation significantly decreased during the second wave (T2) and the fifth wave (T3) compared to T0 with a decline of 0.52 and 0.37 points (cohen’s d equal to 0.26 and 0.19) respectively. At T4, life evaluation recovered to approximately pre-pandemic levels.

#### Positive affect

A statistically significant difference in positive affect was found over time (see Table 3 and Fig. 2b). Positive affect declined significantly during the second lockdown (T2) and the fifth wave (T3) as compared to pre-pandemic (T0) with a decline of 0.18 and 0.42 points (cohen’s d equal to 0.12 and 0.27) respectively. However, at T4 positive affect bounced back and increased significantly with 0.30 points compared to during the fifth wave (T3) (cohen’s d = 0.19).

#### Negative affect

Our results showed no statistically significant differences in negative affect across the five timepoints (see Table 3 and Fig. 2c).

#### Autonomy

There was a statistically significant difference in feelings of autonomy over time (see Table 3 and Fig. 2d). Compared to before the COVID-19 pandemic (T0), feelings of autonomy declined during the second wave (T2) and the fifth wave (T3) with 0.36 and 0.24 points (cohen’s d equal to 0.19 and 0.13) respectively. After restrictions were lifted (T4), feelings of autonomy recovered to pre-pandemic levels.

#### Competence

Our results indicated a statistically significant difference in feelings of competence across the different timepoints (see Table 3 and Fig. 2e). More specifically, feelings of competence were significantly lower during the fifth COVID-19 wave (T3) as compared to before the pandemic (T0) (decline of 0.20 points; cohen’s d = 0.10). Feeling of competence recovered to pre-pandemic levels at T4.

#### Relatedness

A statistically significant difference in feelings of relatedness was found across the five timepoints (see Table 3 and Fig. 2f). Compared to pre-pandemic levels (T0), relatedness increased during the second lockdown (T2) with 0.33 points (cohens’ d = 0.20). At T3, however, feelings of relatedness dropped again to pre-pandemic levels. Our results further show that relatedness increased after the restrictions were lifted (T4) as compared to before the pandemic (T0) with 0.21 points (cohen’s d = 0.13).

#### Loneliness

A statistically significant difference in loneliness was found across the different timepoints (see Table 3 and Fig. 2g). During the second wave (T2) loneliness was increased as compared to before the COVID-19 pandemic (T0) and the first wave (T1) with respectively 0.25 (cohen’s d = 0.11) and 0.32 points (cohen’s d = 0.15). Also, during the fifth wave (T3) loneliness was 0.23 points higher compared to during the first wave (T1) (cohen’s d = 0.11). Loneliness again returned to pre-pandemic levels at T4.

#### Peace of mind

Our results showed a statistically significant difference in peace of mind across the different timepoints (see Table 3 and Fig. 2h). In particular, during the second lockdown (T2), the experienced peace of mind was significantly lower compared to before the pandemic (T0) and during the first wave (T1). A decline of respectively 0.22 (cohen’s d = 0.11) and 0.23 points (cohen’s d = 0.11) was observed. During the fifth wave (T3) peace of mind showed a trend to significant decrease compared to before the pandemic (T0) and during the first wave (T1) (*p *= 0.077 and *p *= 0.068 respectively). Peace of mind recovered to pre-pandemic levels at T4.

### Changes in well-being over time for different age groups

**Figure 3:**
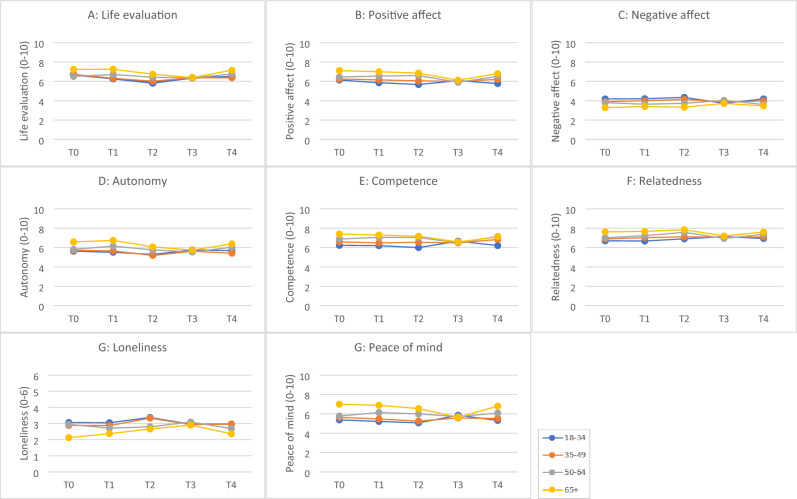
Results of two-way ANOVA predicting (**a**) life evaluation, (**b**) positive affect, (**c**) negative affect, (**d**) autonomy, (**e**) competence, (**f**) relatedness, (**g**) loneliness and (**h**) peace of mind.

Differential evolutions over time for different age groups were investigated using two-way ANOVA. The results of these analyses are shown in Table 4 and Fig. 3a–h.

**Table 4:** Results of two-way ANOVA.

	Age F(3,8064)	Time F(4,8064)	Age*Time F(12,8064)
Life evaluation (0–10)	36.87***	20.33***	4.23***
Positive affect (0–10)	107.40***	20.17***	8.96***
Negative affect (0–10)	57.66***	0.75	5.68***
Autonomy (0–10)	66.51***	15.73***	6.07***
Competence (0–10)	74.22***	5.05***	7.25***
Relatedness (0–10)	52.75***	7.63***	4.25***
Loneliness (0–6)	25.41***	6.73***	2.83**
Peace of mind (0–10)	125.64***	5.95***	10.31***

***p<.001, **p<.01, *p<.05. (independent variables: age, time and age*time; dependent variables: life evaluation, positive affect, negative affect, autonomy, competence, relatedness, loneliness and peace of mind).

#### Life evaluation

There was a significant main effect of time and age. Also, a significant interaction effect was found (see Table 4). In individuals under the age of 50, life evaluation dropped noticeably at T2, whereas for older individuals (65 +), a salient decline in life evaluation occurred later, at T3. In addition, for people of early middle age (35–49) life evaluation did not fully recover to pre-pandemic levels after restrictions were lifted, but the difference in life evaluation at T0 and T4 only showed a trend to significance (*p *= 0.09) (see Fig. 3a).

#### Positive affect

A significant main effect of both time and age were found as well as a significant interaction effect (see Table 4). For people over 50, positive affect declined during the fifth wave (T3) whereas for younger individuals a drop in positive emotions was already noticeable during the second wave (T2). Positive affect returned to pre-pandemic levels for people of middle age (35–64). However, for the youngest individuals (18–34) positive affect did not fully recover at T4. Moreover, this age group experiences the least positive emotions overall (see Fig. 3b).

#### Negative affect

Our results showed a significant main effect for age but not for time. However, the interaction effect was significant (see Table 4). Negative affect gradually increased during the first two waves of the pandemic for the two youngest age groups (18–49). Negative affect then declined at T3 for these individuals. For people over 50, an increase in negative affect is noticeable at T4 (see Fig. 3c).

#### Autonomy

The main effects of time and age were both significant as well as their interaction effect (see Table 4). Our results indicate that autonomy dropped at T2 for the two youngest groups (18–49), whereas for people over 50 a noticeable drop is shown at T3. For all age groups, feelings of autonomy eventually recovered to pre-pandemic levels at T4 (see Fig. 3d).

#### Competence

Our results showed a significant main effect of time and of age. The interaction effect was also significant (see Table 4). For people of early and late middle age, feelings of competence gradually increased over time, whereas for the older individuals (65 +), feelings of competence dropped during the pandemic. For the young adults (ages 18–34) feelings of competence fluctuated but remained stable when we compare T0 and T4. However, feelings of competence in this group are lower as compared to the other groups (see Fig. 3e).

#### Relatedness

The main effects of both time and age were significant, as well as the interaction effect (see Table 4). For individuals over 50, an increase in relatedness was found during the first year of COVID (T0-T2). However, feelings of relatedness in this group dropped at T3, but eventually recovered at T4, after the restrictions were lifted. In individuals under 50, the increase in relatedness is more gradual, with a small setback at T4 for people in the youngest age group (18–34). Feelings of relatedness increased over time in all groups, except for people over 65 (see Fig. 3f).

#### Loneliness

There was a significant main effect of time and age. Also, their interaction was significant (see Table 4). For people under the age of 50, loneliness peaked during the second wave (T2), whereas for older individuals, this peak was delayed and only appeared during the fifth wave (T3). For all age groups loneliness restored to pre-pandemic levels at T4 (see Fig. 3g).

#### Peace of mind

The main effects of time and age were both significant, as well as their interaction (see Table 4). For those under the age of 50, peace of mind dropped notably during the second wave (T2). For people over 50, on the other hand, the decrease in peace of mind is most apparent during the fifth wave (T3). For all age groups, peace of mind eventually returned to pre-pandemic levels at T4, with a small gain for people of late middle age (50–64). Individuals in late adulthood (65 +) furthermore experience more peace of mind than younger subjects (see Fig. 3h).

### Factors related to well-being during the COVID-19 pandemic

As discussed above, we furthermore investigated the role of several explanatory variables—the basic psychological needs (autonomy, competence and relatedness), loneliness and peace of mind—in predicting subjective well-being during the pandemic. Results of the regression models are displayed in Table 5. The adjusted R squared (explained variance) was 39.1% for the dependent variable life evaluation, 38.2% for the dependent variable positive affect, and 47.1% for the predicted variable negative affect.

**Table 5:** Factors contributing to subjective well-being.

	Life evaluation^a^	Positive affect^a^	Negative affect^a^
Time point			
T1	− .03*	− .02	.01
T2	− .07***	− .02	− .00
T3	− .06***	− .07***	− .01
T4	− .03*	− .03*	.00
Autonomy	.10***	.10***	− .04**
Competence	.03(*)	.30***	− .17***
Relatedness	.05***	.01	− .07***
Loneliness	− .14***	− .01	.06***
Peace of mind	.38***	.29***	− .45***
Adjusted R^2b^	39.1%	38.2%	47.1%

All analyses are controlled for: age, gender, educational level, region, occupational status, and equivalised income.

^a^Standardised beta coefficients.

^b^Adjusted R squared represents the total amount of variance explained in the predicted variables (life satisfaction, positive affect, negative affect).

***p ≤ 0.001, **p < 0.01, *p < 0.05, (*)*p *< 0.1. Missing data were not included in the model.

One of the strongest associated factors in all three SWB components was peace of mind, in particular: life evaluation (β = 0.38, *p *< 0.001), positive affect (β = 0.29, *p *< 0.001) and negative affect (β =  − 0.45, *p *< 0.001). Next, loneliness played a crucial independent role in life evaluation (*p *< 0.001) and negative affect (*p *< 0.001), but was not an associated factor with the positive affect component.

With regard to the basic psychological needs, the results show that these needs are also important in explaining SWB, but the importance of their role depends on the SWB component under investigation. In particular, autonomy was significantly related with all SWB components. Competence was a significantly contributing factor of the affective components of SWB, and particularly important in explaining the positive affect component (β = 0.30, *p *< 0.001). With the dependent variable life evaluation, competence only showed a trend towards significance (*p *< 0.10). Finally, relatedness showed significant associations with both life evaluation and negative affect. Nevertheless, this factor was not associated with the positive affect component.

## Discussion

This study aimed to explore the changes in well-being of the Belgian population during the COVID-19 pandemic period. To this end, we used one-way ANOVA to explore differences in well-being over time in repeated cross-sectional representative samples of the Belgian population. We furthermore explored the differential changes in well-being during the pandemic episode for different age groups using moderation analyses. Lastly, by building several multiple linear regression models, we aimed to shed light on the factors contributing to subjective well-being during the pandemic episode.

Overall, our results show that the well-being of the Belgian population decreased during the COVID-19 pandemic. However, all well-being indicators recovered on average to (approximately) pre-pandemic levels after the restrictions were lifted.

For the evaluative component of SWB, we found a significant decline in life evaluation during the second lockdown (T2) and the fifth wave (T3). This decline could be caused by the severe policy measures at the time. Previous research in European countries indeed suggested that policy stringency negatively affects life satisfaction^38^. As such, lockdown and other policy measures should be carefully considered. Thus, contrary to the findings of a recent review^10^, our study shows that life evaluation of the Belgian population did not remain stable during the pandemic.

With regard to the affective component of SWB, individuals living in Belgium reported less positive emotions during the second lockdown (T2) and the fifth wave (T3). The review of Aknin et al.^10^ also showed that positive emotions declined during the first year of the pandemic. Our findings now illustrate that this decline continued the longer the pandemic lasted. Next, with regard to negative affect, we found no differences over time indicating that the Belgian population experienced negative emotions to the same extent during the pandemic. This finding is surprising as previous research repeatedly reported increased distress, anxiety and depression during the COVID-19 pandemic^6,8–10^. However, it could be that diminished positive emotions pose a greater risk for depression as one of the key characteristics of depression is anhedonia (i.e. lack of pleasure)^39^.

Also, significant changes in the basic psychological needs of autonomy, competence and relatedness were found during the COVID-19 pandemic. Autonomy (a sense of psychological freedom) declined significantly at T2 and T3. This finding is not surprising since people’s lives were restricted and failure to comply to the covid-related policy measures was punishable. Competence (sense of effectiveness and mastery) was significantly lower at T3. Relatedness (sense of connection with important others) fluctuated during the pandemic and its aftermath, but an overall positive trend can be noted. Thus, as lockdown measures forcefully decrease the number of social interactions, people seem to tend to invest more time and energy in the interactions that matter the most. The decline in quantity of social interactions, appears to have led to an increase in their quality. Furthermore, social cohesion and solidarity can spark in light of disasters, such as pandemics^40^.

Notwithstanding the increase in relatedness, loneliness increased significantly at T2 and remained elevated at T3. Our results thus contradict those of Aknin et al.^10^ who found that loneliness remained largely stable during the first year of the pandemic. However, the studies regarding loneliness included in their review only covered the first months of the pandemic. It is plausible that people experience a lockdown differently the longer it lasts and gradually become more lonely the longer they are confined, as our results suggest.

We also investigated the pandemic-related changes in peace of mind, an important determinant of mental and general well-being^24,25^. Particularly during the second lockdown (T2), the experienced peace of mind was significantly lower. Peace of mind refers to an emotional state that consists of 2 components: peacefulness (low-arousal positive affect and serenity) and harmony (the process of self-control individuals use to suppress intense feelings)^23^. It is evident that peace of mind declined at this time. The hope for a quick return to normal life was replaced with increasing uncertainty. Also, the broaden-and-build theory^41^ may help to shed light on this decline and subsequent recovery in peace of mind. This theory states that positive emotions bolster people’s strategies to deal with negativity^42^. However, during the pandemic, adults living in Belgium experienced fewer positive emotions according to our results. Hence, their ability to suppress intense feelings weakened and, as such, their peace of mind diminished too. When the experience of positive emotions increased again at T4, a simultaneous upward trend in peace of mind can be observed.

Furthermore, we examined the differential changes in well-being during the pandemic period for various age groups. In line with previous findings^10^, our results indicate that the well-being of younger individuals suffered more during the pandemic period and lockdown measures. In particular, our results suggest that individuals over the age of 50 were more resilient as the downward trend in well-being related to the pandemic period was delayed in this group for several well-being indicators: life evaluation, positive affect, negative affect, autonomy, loneliness and peace of mind. This is in line with previous studies showing that older adults experienced less negative effects of the pandemic on their psychological well-being and stress^43,44^. This resilience could be related to more adaptive coping mechanisms in this group compared to younger individuals^44^. Our results also show that compared to older adults, younger individuals experience less peace of mind which appears to be an important predictor of all SWB components. However, more research on the mechanisms explaining the age differences in pandemic-related changes in well-being is warranted. Our finding that younger individuals experienced worse changes in well-being during the COVID-19 pandemic period could also indicate a more general trend of declining well-being in younger generations. For instance, a recent study conducted in Germany has shown an increase in depressive symptoms for younger generations^45^. According to our results, this downward trend might have been magnified during the COVID-19 pandemic period. More research on factors protecting well-being and potential harmful factors is thus warranted, for younger individuals as well as across the lifespan.

Lastly, we investigated the role of several explanatory variables—autonomy, competence, relatedness, loneliness and peace of mind—in predicting subjective well-being during the pandemic. Our results show that especially peace of mind is strongly related to all components of SWB. This finding is in line with previous research indicating that peace of mind is an important determinant of mental and psychological well-being^24,25^. With regard to the affective components of subjective well-being, competence (a sense of mastery and efficacy) was a strong contributing factor.

### Practical implications and recommendations

#### Recommendation 1: Support research on the long-term consequences of the pandemic on well-being and means to restrengthen the well-being of nations

Our results clearly illustrate a negative trend in the general well-being of the Belgian population related to the COVID-19 pandemic and its policy measures. Fortunately, well-being largely recovered after the restrictions were lifted showing the resilience of a nation. This finding is remarkable in light of the current crises Europe is facing (i.e. rising energy prices, inflation and the war in Ukraine) and illustrates the importance of social connection for a nation’s well-being. Previous research has indeed shown that social connection is a crucial ingredient for subjective well-being^46^. Also, research has shown that during the pandemic social support had a positive effect on peace of mind^47^, a strong predictor of all SWB well-being components according to our findings. The pandemic-related changes in well-being may be small in magnitude as all found cohen’s d range between 0.10 and 0.30, but should not be underestimated. Therefore, and as previously recommended by Aknin et al.^10^, we call for more research on the long-term consequences of the pandemic on the general well-being of nations. Future research should also explore how to restrengthen mental and subjective well-being. Furthermore, we urge governments to act upon such research and to actively invest in and support the well-being of their nations.

#### Recommendation 2: Invest in accessible mental health care services

A crisis, like the COVID-19 pandemic, reveals the weaknesses of a system. Access to adequate mental health care services is one of those weaknesses. A study by Rens et al.^48^ highlighted the issue of unmet mental health needs in the general population in Belgium. Moreover, nearly half of the patients in Belgium had to wait one month or more for a first contact in 2017^49^. For children and adolescents this issue is even worse with waiting times as long as six months or longer, in both the private sector and crisis care^50^. It is thus clear that mentally ill individuals do not have equal access to evidence-based treatment than those who are physically ill. Therefore, we urge governments to invest in the accessibility of mental health care services in the aftermath of the pandemic. The Belgium government, for instance, has recently increased the budget for the reimbursement of primary psychological care. Although a good starting point, these measures may not be sufficient. In this new convention adults will only be reimbursed for 8 individual sessions or 5 group sessions. In most cases, this amount will be insufficient to enable durable change. In addition, by only reimbursing a fixed number of sessions, it is indirectly communicated to patients that they should be “cured” by this time. As such, they might become demotivated if notable change takes longer.

#### Recommendation 3: Policy measures should consider all aspects of health

Public health is concerned with preventing disease, prolonging life and promoting health through organized societal efforts^51^. The WHO recognizes that health not only encompasses a physical aspect, but also underlines the importance of mental and social well-being in its definition of health^52^. Nevertheless, policy measures during the COVID-19 pandemic were mainly focused on securing physical health and preventing a healthcare system collapse^3,4^, thereby paying less attention to the impact of the pandemic on other aspects of health. Yet, the number of people whose mental health is affected during epidemics might outweigh the number of people affected by the infection itself^53^. According to the 2022 Health at a Glance report for instance, the number of young people reporting depressive symptoms has more than doubled during the COVID-19 crisis in several EU countries^54^. Also, our results showed that the overall well-being of a nation declined during the pandemic. Therefore, we strongly recommend policymakers to consider all aspects of health when installing measures to tackle (future) pandemics. Policymakers are furthermore advised to timely adapt their strategies so people are no longer confined than absolutely necessary^4^.

#### Recommendation 4: Policymakers should be more attentive to the mental health needs of younger members in their nations, especially during pandemics

Our results indicate that younger individuals are more susceptible to negative changes in well-being during the pandemic period. Also, mental health problems are more prevalent in younger adults^55^. When implementing measures, policymakers are thus advised to protect the well-being of the younger members of their nations as much as possible. It is of great importance to prevent mental health problems given their great burden not only on the individual, but also on society^56^. In addition, strengthening resilience and mental well-being in this younger group is of key importance, and should ideally start early on. Indeed, mental health in childhood and adolescence predicts mental health in adulthood^57^. Therefore, governments should enable well-being promotion early on by including positive psychology in education. The school is indeed a valuable setting to implement positive psychology interventions targeting well-being for both children^58,59^ and adolescents^60^.

### Strengths and limitations

To better understand the context of these findings, some strengths and weaknesses should be mentioned. A first strength is that this study had a notably large and representative sample of the Belgian population at all five timepoints. A second strength is that many variables and concepts potentially related to SWB were questioned allowing to better understand the pandemic-related changes in well-being in a very broad sense. Third, because of the first measurement point being right before the start of the pandemic in Belgium, the results of this study contribute to new evidence on the consequences of the pandemic period and its associated policy measures by investigating the long-term changes in the well-being of the Belgian population during the pandemic episode. Fourth, based on our results, we were able to formulate valuable policy recommendations and a clear call for future research.

Our study also had some limitations. First, to investigate the long-term pandemic-related changes in well-being, this study used repeated cross-sectional surveys instead of the golden standard of longitudinal research. Nevertheless, our results are based on large representative samples of the Belgian population. Moreover, and as expected of representative samples, these samples had similar characteristics at all different timepoints. As such, we are convinced we can draw valid conclusions about the long-term changes in the well-being of the Belgian population related to the pandemic period. Second, public health studies such as this study can suffer from a sampling bias as individuals with worse health and well-being are less likely to participate and more likely to drop out^61,62^. As such, if such sampling bias occurred, this would mean that the true levels of well-being in the Belgian population are in fact lower than those reported in our study. Analyses of association, however, appear to be less affected by a sampling bias^61^. In addition, our study may have suffered from other forms of sampling bias. As an online questionnaire was used, only people who have access and who are familiar with internet could participate. Also, as people were recruited via a research and consulting company, we have no data on the non-response. Notwithstanding the fact sample bias might have occurred in our study, our samples at the various timepoints were representative for the Belgian population and weighted based on several sociodemographic characteristics such as age, gender, educational level, occupational status and region. Third, although it was certainly a major strength that many concepts were questioned, this approach resulted in the necessity to shorten or slightly adapt some measurements in order to limit the time to fill out the questionnaire and avoid response bias. As a result, peace of mind was no longer assessed using the strictly validated measurement of Lee et al.^23^. Nonetheless, all Cronbach’s alpha’s were still sufficient. Also, when Dutch or French versions were not available in the literature, we had to translate them using a forward back translation method. Lastly, we only used self-report questionnaires which can induce social desirability bias. However, social desirability bias should have remained limited because the questionnaire was strictly anonymous. Nevertheless, future research could benefit from using additional measurement methods to obtain information about SWB and other well-being indicators.

## Conclusion

The well-being of the Belgian population decreased during the COVID-19 pandemic, especially during the second and fifth wave. After the restrictions were lifted, all well-being indicators recovered to (approximately) pre-pandemic levels, showing the resilience of a nation. Our results further indicate that younger individuals are more susceptible to negative well-being changes related to the pandemic. Moreover, according to our findings, peace of mind is an important predictor of all SWB components (life evaluation, positive affect and negative affect) in times of a pandemic. These insights should guide policymakers in governing a (future) pandemic and its aftermath.

## Data Availability

The data supporting this article can be made available from the corresponding author upon reasonable request.
